# HIF-2α-dependent TGFBI promotes ovarian cancer chemoresistance by activating PI3K/Akt pathway to inhibit apoptosis and facilitate DNA repair process

**DOI:** 10.1038/s41598-024-53854-y

**Published:** 2024-02-16

**Authors:** Sijia Ma, Jia Wang, Zhiwei Cui, Xiling Yang, Xi Cui, Xu Li, Le Zhao

**Affiliations:** 1 Department of Obstetrics and Gynecology, The First Affiliated Hospital of Xi’an Jiaotong University, Xi’an, 710061 Shaanxi People’s Republic of China; 2 Center for Translational Medicine, The First Affiliated Hospital of Xi’an Jiaotong University, Xi’an, 710061 Shaanxi People’s Republic of China; 3 Key Laboratory for Tumor Precision Medicine of Shaanxi Province, The First Affiliated Hospital of Xi’an Jiaotong University, Xi’an, 710061 Shaanxi People’s Republic of China; 4 Precision Medicine Center, The First Affiliated Hospital of Xi’an Jiaotong University, Xi’an, China

**Keywords:** Ovarian cancer, Hypoxia, Chemoresistance, Akt, DNA repair, Cancer, Cell biology, Computational biology and bioinformatics, Developmental biology, Drug discovery, Molecular biology, Biomarkers, Diseases, Medical research, Molecular medicine, Oncology, Pathogenesis, Risk factors, Mathematics and computing

## Abstract

Hypoxia-mediated chemoresistance plays a crucial role in the development of ovarian cancer (OC). However, the roles of hypoxia-related genes (HRGs) in chemoresistance and prognosis prediction and theirs underlying mechanisms remain to be further elucidated. We intended to identify and validate classifiers of hub HRGs for chemoresistance, diagnosis, prognosis as well as immune microenvironment of OC, and to explore the function of the most crucial HRG in the development of the malignant phenotypes. The RNA expression and clinical data of HRGs were systematically evaluated in OC training group. Univariate and multivariate Cox regression analysis were applied to construct hub HRGs classifiers for prognosis and diagnosis assessment. The relationship between classifiers and chemotherapy response and underlying pathways were detected by GSEA, CellMiner and CIBERSORT algorithm, respectively. OC cells were cultured under hypoxia or transfected with HIF-1α or HIF-2α plasmids, and the transcription levels of TGFBI were assessed by quantitative PCR. TGFBI was knocked down by siRNAs in OC cells, CCK8 and in vitro migration and invasion assays were performed to examine the changes in cell proliferation, motility and metastasis. The difference in TGFBI expression was examined between cisplatin-sensitive and -resistant cells, and the effects of TGFBI interference on cell apoptosis, DNA repair and key signaling molecules of cisplatin-resistant OC cells were explored. A total of 179 candidate HRGs were extracted and enrolled into univariate and multivariate Cox regression analysis. Six hub genes (TGFBI, CDKN1B, AKAP12, GPC1, TGM2 and ANGPTL4) were selected to create a HRGs prognosis classifier and four genes (TGFBI, AKAP12, GPC1 and TGM2) were selected to construct diagnosis classifiers. The HRGs prognosis classifier could precisely distinguish OC patients into high-risk and low-risk groups and estimate their clinical outcomes. Furthermore, the high-risk group had higher percentage of Macrophages M2 and exhibited higher expression of immunecheckpoints such as PD-L2. Additionally, the diagnosis classifiers could accurately distinguish OC from normal samples. TGFBI was further verified as a specific key target and demonstrated that its high expression was closely correlated with poor prognosis and chemoresistance of OC. Hypoxia upregulated the expression level of TGFBI. The hypoxia-induced factor HIF-2α but not HIF-1α could directly bind to the promoter region of TGFBI, and facilitate its transcription level. TGFBI was upregulated in cisplatin-sensitive and resistant ovarian cancer cells in a cisplatin time-dependent manner. TGFBI interference downregulated DNA repair-related markers (p-p95/NBS1, RAD51, p-DNA-PKcs, DNA Ligase IV and Artemis), apoptosis-related marker (BCL2) and PI3K/Akt pathway-related markers (PI3K-p110 and p-Akt) in cisplatin-resistant OC cells. In summary, the HRGs prognosis risk classifier could be served as a predictor for OC prognosis and efficacy evaluation. TGFBI, upregulated by HIF-2α as an HRG, promoted OC chemoresistance through activating PI3K/Akt pathway to reduce apoptosis and enhance DNA damage repair pathway.

## Introduction

Ovarian cancer (OC) remains one of the most lethal malignancies affected gynecologic reproductive systems, accounting for 5% estimated deaths in females. Despite the development of OC treatment strategies, the overall 5-year survival rate for OC patients remains only 49% for all races and 24% for advanced patients^1^. Establishing molecular models using effective biomarkers for diagnosis and survival prediction is vital in optimizing patient stratification and medical decision-making.

Hypoxia is an inherent characteristic of solid malignancies as the vascular nutrient is insufficient^2^. Emerging studies have demonstrated that the pivotal roles of hypoxia on tumor progression, metastasis, chemoresistance and immunosuppression^3,4^. For ovarian cancer, the oxygen supply to ovarian cancer is severely impaired by ascites, which exacerbates hypoxia dependency^5^. Therefore, investigating the physiological and pathological effects of hypoxia, understanding its molecular mechanisms sustaining cancer development and chemotherapy response and identifying effective targets are essential for early detection, diagnosis, and medical intervention of OC.

Currently, the analysis of molecular mechanisms based on comprehensive bioinformatics is becoming increasingly imperative in cancer researches^6,7^. Recently, attentions have been focused on the identification of novel signatures in cancer early diagnosis^8^, molecular typing^9^, medical decision-making^10^ and prognosis prediction^11^ with the application of comprehensive bioinformatics. Specifically, the comprehensive analysis of hypoxia-related signature for diagnosis prediction, immune microenvironment and prognosis assessment were explored in hepatocellular carcinoma^12^, cervical carcinoma^13^, gastric carcinoma^14^ and ovarian carcinoma^15^. However, there is still lacking in effective hypoxia-related diagnostic and therapeutic targets for OC.

In this report, an integrated evaluation of the expression profiles of hypoxia-related genes (HRGs) was conducted to assess the function of HRGs on OC progression, prognosis and chemoresistance. TGFBI, a key hub HRGs, was validated as a HIF-2α-responsive gene and promoted OC cisplatin resistance via activating PI3K/Akt pathway.

## Results

### Construction of hypoxia-related prognostic model in OC

**Figure 1:**
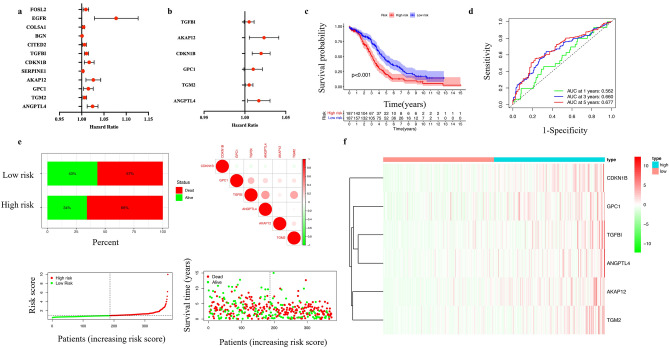
Prognostic value of the hypoxia risk signature in OC. (**a**) The univariate Cox regression analysis of 12 hypoxia-related genes. (**b**) The univariate Cox regression analysis of 6 hypoxia-related genes. (**c**) The survival probability of prognosis model based on the risk score in training cohort. (**d**) The area under the curve of prognosis model based on the risk score in training cohort. (**e**) Distribution of risk score, OS, and OS status of the 6 prognostic hypoxia risk gene signatures in the training cohort. (**f**) The heatmap of the 6 prognostic hypoxia risk gene signatures in the training cohort.

To better understand the imperative function of HRGs in oncogenesis of OC, 200 genes associated with the hallmark gene sets of hypoxias were retrieved from Molecular Signatures Database. The prognostic function of 179 hub HRGs in OC patients was explored (Supplementary Fig. 1). Based on the univariate Cox regression analysis, 12 hub HRGs were found to be significantly correlated with patients’ overall survival (OS), including FOS like 2, AP-1 transcription factor subunit (FOSL2), epidermal growth factor receptor (EGFR), collagen type V alpha 1 chain (COL5A1), biglycan (BGN), Cbp/p300 interacting transactivator with Glu/Asp rich carboxy-terminal domain 2 (CITED2), transforming growth factor beta-induced protein (TGFBI), cyclin-dependent kinase inhibitor 1B (CDKN1B), serpin family E member 1 (SERPINE1), A-kinase anchoring protein 12 (AKAP12), glypican 1 (GPC1), transglutaminase 2 (TGM2) , and angiopoietin-like 4 (ANGPTL4) (Fig. 1a). In the multivariate Cox regression analyses, 6 hypoxia-related genes including TGFBI, AKAP12, CDKN1B, GPC1, TGM2 and ANGPTL4 were chosen to build the predictive model (Fig. 1b). Based on the coefficients obtained from the Cox regression algorithm, the risk scores of the training and validation cohorts were calculated, respectively. Patients were divided into high-risk and low-risk groups based on median of the risk score. The prognosis Index of prognosis model was showed in Supplementary Table 1.

$$\begin{aligned} Prognosis\;Index & = \left( {0.005{\text{*expression}}\;{\text{level}}\;{\text{of}}\;{\text{TGFBI}}} \right) + \left( {0.020{*}\;{\text{expression}}\;{\text{level}}\;{\text{of}}\;{\text{CDKN}}1{\text{B}}} \right) \\ & \quad + \left( {0.023{\text{*expression}}\;{\text{level}}\;{\text{of}}\;{\text{AKAP}}12} \right) + \left( {0.010{\text{*expression}}\;{\text{level}}\;{\text{of}}\;{\text{GPC}}1} \right) \\ & \quad + \left( {0.005{\text{*expression}}\;{\text{level}}\;{\text{of }}\;{\text{TGM}}2} \right) + \left( {0.017{\text{*expression}}\;{\text{level}}\;{\text{of}}\;{\text{ANGPTL}}4} \right) \\ \end{aligned}$$

The OS of the high-risk group was shorter in the training cohorts (Fig. 1c). The AUC was 0.562 at 1 year, 0.660 at 3 years, and 0.677 at 5 years in the training cohort, respectively, indicating a higher predictive value with longer follow-up (Fig. 1d).

The distribution of status, gene correlation, risk scores and survival time of the 6 hub HRGs in the training cohort was displayed in Fig. 1e. The high-risk group had higher mortality compared with low-risk group. And the 6 HRGs were highly expressed in the high-risk group as showed in heatmap (Fig. 1f), indicating that patients in the high-risk group tended to develop hypoxic microenvironments.

### Identification of hypoxia-related signaling pathways in OC

GSEA results (Hallmarks analysis, KEGG analysis and GO analysis) showed that signaling pathways correlated with oncogenesis and chemoresistance were significantly enriched in the high hypoxia risk group (Supplementary Fig. 2), including UV response, apical junction, focal adhesion, receptor interaction, epithelial mesenchymal transition signaling pathway, hypoxia, DNA repair and homologous recombination, indicating that the hub HRGs may contribute to chemoresistance in OC via activating DNA damage and repair pathway.

### Immunity analysis between high and low hypoxia risk groups in OC

The assessment of hypoxia-related risk signals in the immune microenvironment was explored through CIBERSORT. The heatmap of all immune cells showed that patients with higher hypoxia risk score had significantly lower percentages of B cells memory and higher fractions of Microphages M2 in the training cohort (Supplementary Fig. 3a) and significantly higher percentages of B cells memory and lower fractions of Mast cells resting (Supplementary Fig. 3b). Immune checkpoints such as PD-L2, immune-related genes, such as CXCR3, CCL20, CCL19, CXCL16, CCR5, CXCL11, CX3CL1, CXCL9 and CXCL10 were upregulated in the high-risk group (Supplementary Fig. 4a), which was also validated in the validation cohort (Supplementary Fig. 4b).

### Construction of hypoxia-related diagnosis model in OC

The diagnostic prediction model was established to effectively predict OC based on the six-hub hypoxia-related genes (TGFBI, AKAP12, GPC1, TGM2 and ANGPTL4). The binary FR models were as follows:

$$\begin{aligned} \log it\left( {P1} \right) & = 4.680 - 0.002{\text{*expression}}\;{\text{level}}\;{\text{of}}\;{\text{TGFBI}} \\ \log it\left( {P2} \right) & = 4.138 - 0.003{\text{*expression}}\;{\text{level}}\;{\text{of}}\;{\text{TGFBI}} + 0.021*{\text{expression}}\;{\text{level}}\;{\text{of}}\;{\text{GPC}}1 \\ \log it\left( {P3} \right) & = 4.879 - 0.003{\text{*expression}}\;{\text{level}}\;{\text{of}}\;{\text{TGFBI}} - 0.006 \\ & \quad {\text{*expression}}\;{\text{level}}\;{\text{of}}\;{\text{AKAP}}12 + 0.022*{\text{expression}}\;{\text{level}}\;{\text{of}}\;{\text{GPC}}1 \\ \log it\left( {P4} \right) & = 4.699 - 0.005{\text{*expression}}\;{\text{level}}\;{\text{of}}\;{\text{TGFBI}} - 0.007{\text{*expression}}\;{\text{level}}\;{\text{of}}\;{\text{AKAP}}12 + 0.022 \\ & \quad *{\text{expression}}\;{\text{level}}\;{\text{of}}\;{\text{GPC}}1 + 0.022*{\text{expression}}\;{\text{level}}\;{\text{of}}\;{\text{TGM}}2 \\ \end{aligned}$$

**Figure 2:**
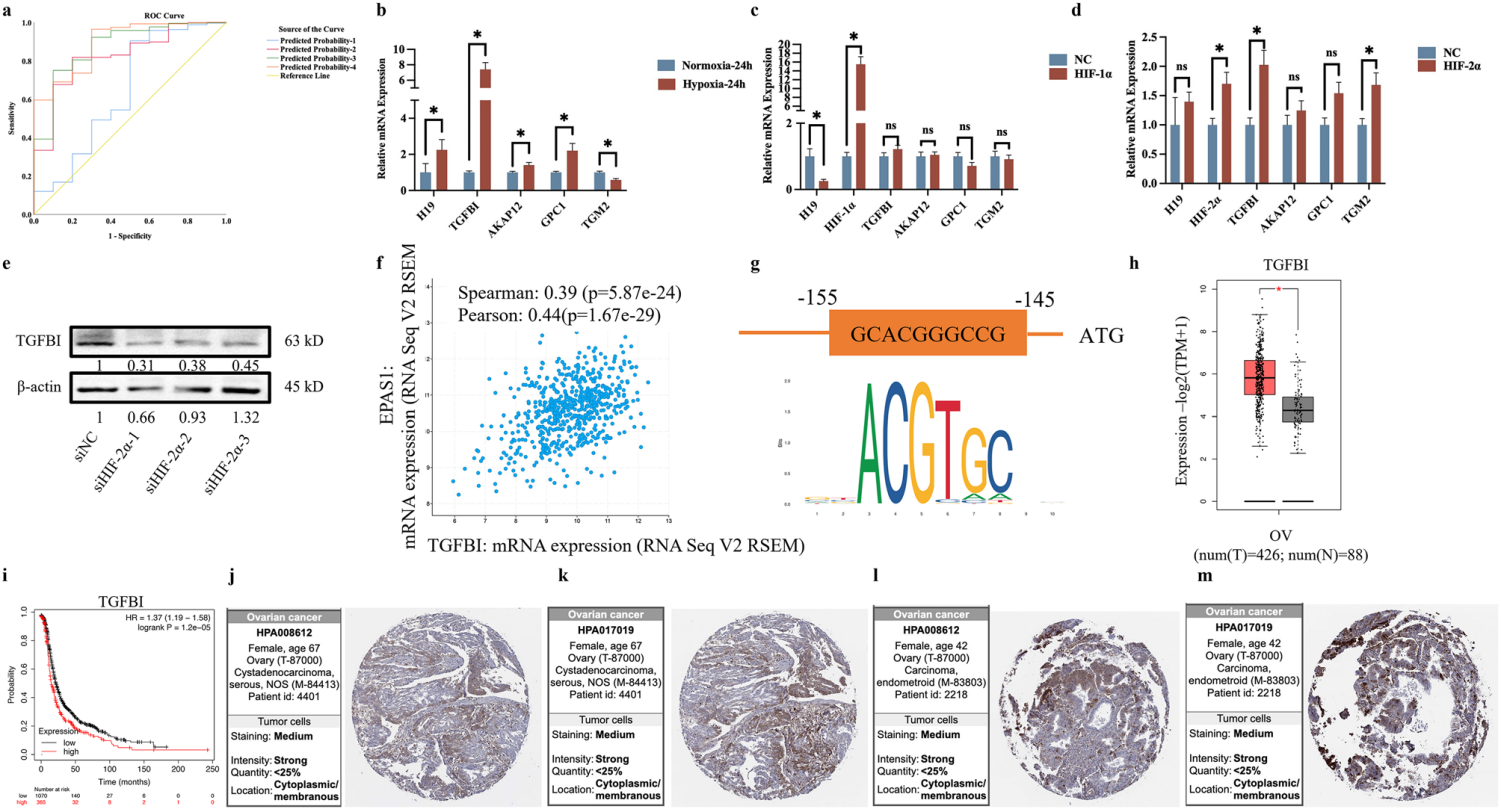
The role of hypoxia-related genes in hypoxic condition. (**a**) The area under the curve for four diagnostic models. (**b**) The mRNA expression level of TGFBI, AKAP12, GPC1 and TGM2 in normoxia and hypoxia condition. (**c**) The mRNA expression level of TGFBI, AKAP12, GPC1 and TGM2 after transfected with HIF1a-plasmid. (**d**) The mRNA expression level of TGFBI, AKAP12, GPC1 and TGM2 after transfected with HIF2a-lentivirus. (**e**) The protein expression level of TGFBI after transfected with siHIF2a. (**f**) The Pearson and Spearman correlation analysis of TGFBI and EPAS1. (**g**) The promotor region of TGFBI and binding motif of HIF2a. (**h**) Expression level of TGFBI in ovarian cancer and normal tissues. (**i**) K–M survival curves of TGFBI in ovarian cancer. (**j**–**m**) Validation of TGFBI at the translational level using the Human Protein Atlas (HPA) database (IHC).

The AUCs of the predicted probability 1, 2, 3 and 4 were 0.645, 0.826, 0.875 and 0.893, respectively (Supplementary Table 2). The Variables in the equation of the four diagnostic models were shown in Supplementary Table 3. Figure 2a showed the AUC of the four predicted models.

### The validation of hub HRGs in hypoxia condition

To validate hub HRGs in hypoxia condition, the hypoxia-sensitive human ovarian cancer cell, SKOV3, was cultured under normoxia and consistent 1% O_2_ hypoxia condition for 24 h, respectively. The qRT-PCR result showed that the mRNA levels of four hub HRGs (TGFBI, AKAP12, GPC1 and TGM2) were significantly higher in hypoxia condition than normoxia condition, validating these four genes were hypoxia-responsive genes (Fig. 2b). In order to determine which hypoxia factor contributed to their expression, HIF-1α and HIF-2α were overexpressed separately. After transfected with HIF-1α, the mRNA levels of the four genes were insignificantly changed, indicating that the four genes were not HIF-1α-responsive (Fig. 2c). But TGFBI was significantly higher in HIF-2α-overexpressed cells (Fig. 2d). And in HIF-2α-knocked down cells, the protein levels of TGFBI were subsequently reduced (Fig. 2e), indicating that TGFBI was transcriptionally regulated by HIF-2α. Additionally, the correlation analysis of HIF-2α and TGFBI demonstrated that the mRNA levels of HIF2α and TGFBI were positively correlated with Spearman 0.39, Pearson 0.44, P < 0.05 (Fig. 2f). The promoter region of TGFBI (specifically -155- -145 “GCACGGGCCG”) was predicted as the binding motif of HIF-2α, indicating that HIF-2α may directly contribute to the high expression of TGFBI (Fig. 2g). Moreover, the mRNA level of TGFBI was significantly higher in ovarian cancer tissues than in normal tissues (Fig. 2h), and the higher level of TGFBI, the poorer prognosis of ovarian cancer patients (Fig. 2i). Furthermore, the protein level of TGFBI was strongly expressed in OC tissues using standard IHC labeling collected from HPA (Figs. 2j–m).

### The role of TGFBI in the regulation of OC progression and chemotherapy in vitro

**Figure 3:**
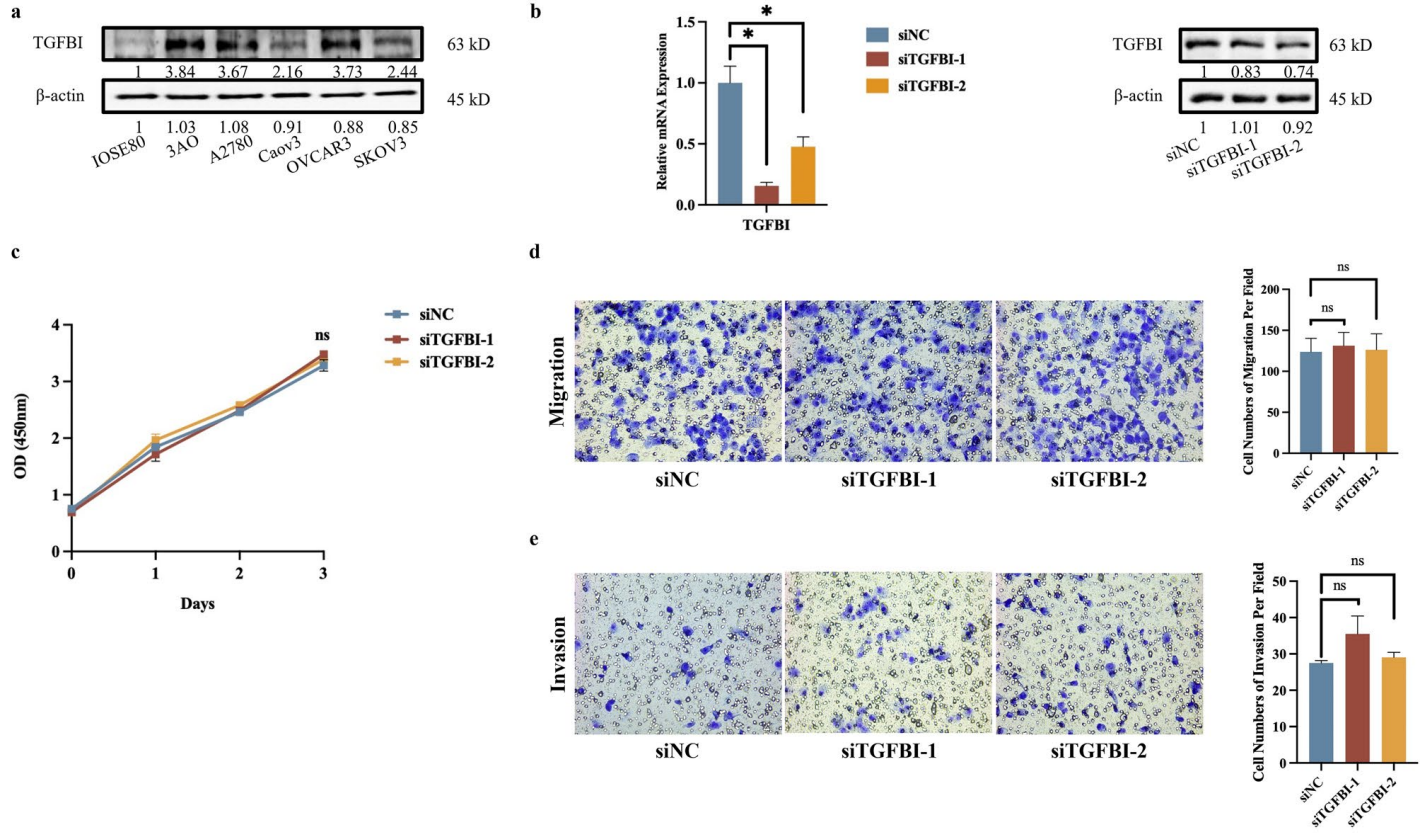
The role of TGFBI in ovarian cancer proliferation, migration and invasion. (**a**) The protein expression level of TGFBI in normal ovarian cell line and 5 ovarian cancer cell lines. (**b**) The mRNA and protein expression level of TGFBI after knocking down by siRNA. The cell proliferation (**c**), migration (**d**) and invasion (**e**) after knocking down TGFBI in 3AO cell line. The blots have been cropped to improve the conciseness and clarity of the display.

In order to explore the role of TGFBI in the regulation of OC, several experiments in vitro were conducted. First of all, as shown in Fig. 3a, the protein level of TGFBI was higher in 3AO than in normal human ovarian epithelial cell line IOSE80 and four other OC cell lines (A2780, Caov3, OVCAR3 and SKOV3). TGFBI interference by siRNAs in 3AO brought about no significant changes in cell viability, migration and invasion (Fig. 3b–e).

**Figure 4:**
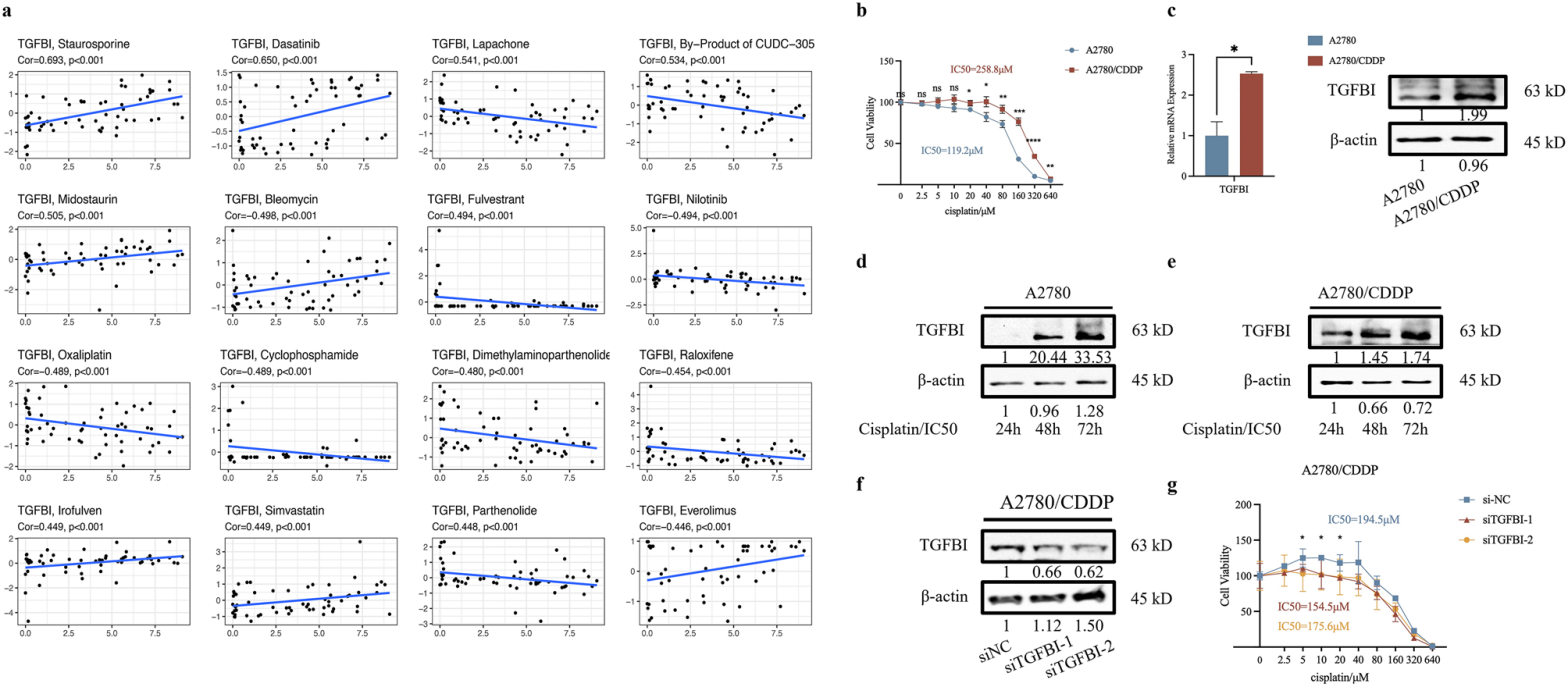
The role of TGFBI in ovarian cancer chemoresistance. (**a**) Evaluation of TGFBI sensitivity to chemotherapy drugs on tumor. (**b**) Cell survival between A2780 and A2780/CDDP followed by the concentration gradient stimulation of cisplatin. (**c**) The mRNA and protein expression level of TGFBI in cisplatin-sensitive ovarian cancer cell line-A2780 and cisplatin-resistant ovarian cancer cell line-A2780/CDDP. (**d**) The protein expression level of TGFBI after time gradient of cisplatin in A2780 (IC_50_ as 119.2 μM). (**e**) The protein expression level of TGFBI after time gradient of cisplatin in A2780/CDDP (IC_50_ as 258.8 μM). (**f**) The protein expression level of TGFBI after knocking down by siRNA in A2780/CDDP. (**g**) Cell survival after A2780/CDDP were transfected with siRNA, followed by the concentration gradient stimulation of cisplatin (0, 2.5, 5, 10, 20, 40, 80, 160, 320 and 640 μM).

In view of a potential role of TGFBI in platinum-based therapy revealed by the Evaluation of drug sensitivity (Fig. 4a), TGFBI expression level was examined in cisplatin-resistant ovarian cancer cell line A2780/CDDP and cisplatin-sensitive ovarian cancer cell line A2780 (as showed in Fig. 4b, IC_50_ for A2780 was 119.2 μM, IC_50_ for A2780/CDDP was 258.8 μM), the results showed that TGFBI was elevated at both mRNA and protein levels in A2780/CDDP cells (Fig. 4c). And with the time gradient stimulation of cisplatin, the protein expression levels of TGFBI were elevated in A2780 and A2780/CDDP cells (Fig. 4d and e). Therefore, TGFBI was interfered by siRNAs in A2780/CDDP (Fig. 4f) and the IC_50_ values for A2780/CDDP cells transfected with siNC and siTGFBIs were 194.5 μM, 154.5 μM and 175.6 μM, respectively, indicating that TGFBI contributed to cisplatin-resistance in OC (Fig. 4g).

### TGFBI promoted chemoresistance through inhibiting apoptosis and facilitating DNA damage repair via activating PI3K/Akt signaling pathway

**Figure 5:**
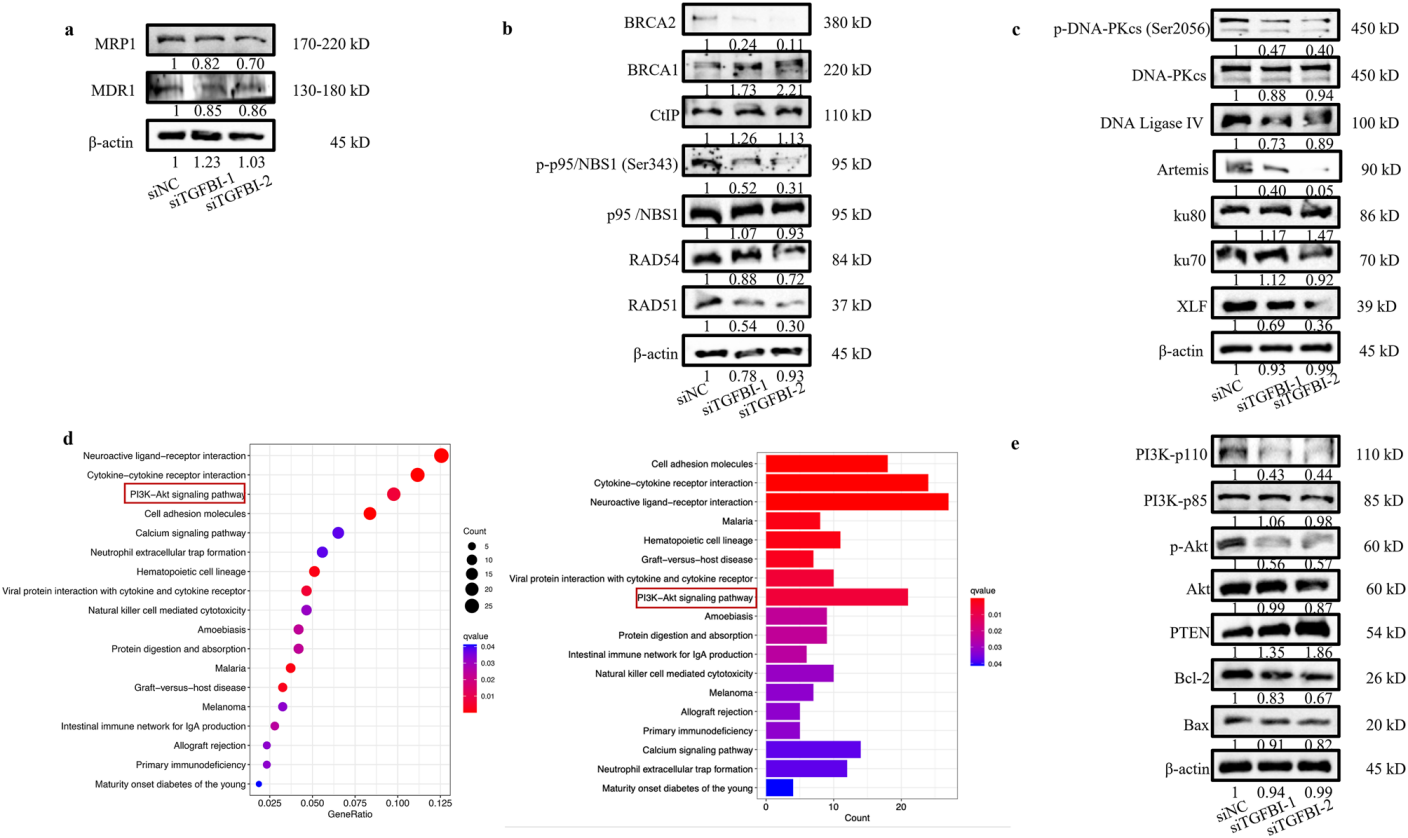
TGFBI promoted chemoresistance through inhibiting apoptosis and facilitating DNA damage repair via activating PI3K/Akt signaling pathway. (**a**) The protein expression level of MRP1 and MDR1 in A2780/CDDP after TGFBI knockdown. (**b**) The protein expression level of Homologous Recommendation DNA repair related markers in A2780/CDDP after TGFBI knockdown. (**c**) The protein expression level of Non-Homologous End Joining (NHEJ) DNA repair related markers in A2780/CDDP after TGFBI knockdown. (**d**) The KEGG enrichment pathway analysis of TGFBI-related differentially expressed genes in OC. (**e**) The protein expression level of PTEN, PI3K/p110, PI3K/p85, p-Akt, Akt, BCL2, Bax and Beclin1 in A2780/CDDP after TGFBI knockdown.

In order to explore the underlying mechanism by which TGFBI promoted chemoresistance in OC, we further investigated the effect of TGFBI on the expression of multidrug resistant-related markers and DNA damage repair pathway in A2780/CDDP cells. The Western blot results showed that suppression of TGFBI downregulated MRP1 and MDR1 (Fig. 5a). TGFBI knockdown reduced the protein levels of BRAC2, p-p95, RAD51, p-DNA-PKcs, DNA Ligase IV and Artmis, proving that TGFBI promoted chemoresistance via the combination of Homologous Recommendation DNA repair (Fig. 5b) and Non-Homologous End Joining (NHEJ) DNA repair (Fig. 5c). KEGG analysis indicated the involvement of TGFBI in PI3K/Akt pathway (Fig. 5d). In siTGFBIs-transfected A2780/CDDP cells, PI3K-p110 and p-Akt were reduced while Akt negative regulator PTEN was increased. Additionally, the anti-apoptotic protein Bcl-2 was decreased while pro-apoptotic protein Bax remained unchanged with TGFBI inhibition (Fig. 5e). Taken together, TGFBI activated PI3K/Akt pathway to inhibit apoptosis and facilitate DNA repair to promote chemoresistance in OC.

## Discussion

Ovarian cancer (OC) is one of the deadly gynecological malignancies in the worldwide, and the progression of OC is a complicated process regulated by various factors^16^. The prognosis of OC patients remains poor in spite of the development of emerging therapeutic options for OC^17^. Currently, high-throughput sequencing complimented with integrated big-data analysis have gradually become more and more important applications for medical research, which can effectively identify biomarkers for diagnosis, clinical stratification, prognosis and recurrence monitoring et al.^18^. Bioinformatic analysis focusing on one biophysical, biochemical or biologic feature of OC will help to explore key targets in OC progression.

Hypoxia is a prominent characteristic of malignant tumors, especially in OC^19^. It was found that hypoxic environment was involved in the aggressive progression of OC with significantly poor prognosis^20^. Nevertheless, the detailed mechanism of hypoxia in OC remains unclear^15^. Hence, exploring the role and underlying mechanism of hypoxia in OC may offer opportunities for potential therapeutic purposes. In this study, six hypoxia-related genes (TGFBI, AKAP12, CDKN1B, GPC1, TGM2 and ANGPTL4) were identified for their correlations with the prognosis of OC and four of them (TGFBI, AKAP12, GPC1 and TGM2) were chosen for prediction model of OC. TGFBI, also known as βIGH3, has been demonstrated to play roles in regulating cell adhesion and the pathogenesis of human disease^21,22^. AKAP12 is involved in promoting colon cancer metastasis via HDAC6-dependent AKAP12 deacetylation and ubiquitination mediated degradation^23^. CDKN1B, encoding for the CDK inhibitor p27^kip1^, has been confirmed mutant in various cancers including breast^24^, prostate cancer^25^ and small intestine neuroendocrine tumors^26^, of which the mutation and copy number variation are associated with tumor aggressiveness^27^. GPC1, as well as glypican-1, is specifically enriched on cancer-cell-derived exosomes and may serve as a potential non-invasive diagnostic and screening tool to detect early stages of pancreatic cancer^28^. TGM2 transcriptionally activates FN1 by promoting nuclear factor kappa B (NFκB) p65 nuclear translocation, ultimately promoting PTC invasion/metastasis^29^. ANGPTL4, plays a critical role in regulating reactive oxygen species (ROS) production, which might provide new targets for improving outcomes in patients with hyperlipidemia-associated CRC metastasis^30^. Our results showed that these six hub HRGs were independent factors for evaluating the prognosis of OC with great predictive performance. According to the prognosis Index, we divided the OC patients into two clusters (high- and low-risk groups) and compared the differences between the two groups. It should be pointed out that the high-risk group had a higher PD-L2 expression, indicating that high-risk patients were more likely to benefit from immunocheckpoint inhibitor therapy. More importantly, the diagnostic model based on the four HRGs (TGFBI, AKAP12, GPC1 and TGM2) presented a higher accuracy and sensitivity and could accurately and effectively distinguish OC from normal ovary.

Much attention has been paid on the role of hypoxia in regulating the immune microenvironment in tumor progression and metastasis^12^. Several studies have demonstrated that hypoxia can interfere with tumor-associated immune cells to enhancing proliferation, differentiation, vessel growth and distant metastasis of tumors^31–33^. However, a number of inhibitory pathways, known as immunocheckpoints, were responsible for suppressing this anti-tumor response in the tumor microenvironment^34^. Our results demonstrated that the high-risk group had a higher fractions of B memory cells and M2 macrophages and higher expression levels of immune checkpoints like PD-L2 and immune-related genes including CCL19, CCR5, CX3CL1 and CXCL9. These results highlighted the pivotal role of immunotherapy for high-risk OC patients based on the expression levels of HRGs.

**Figure 6:**
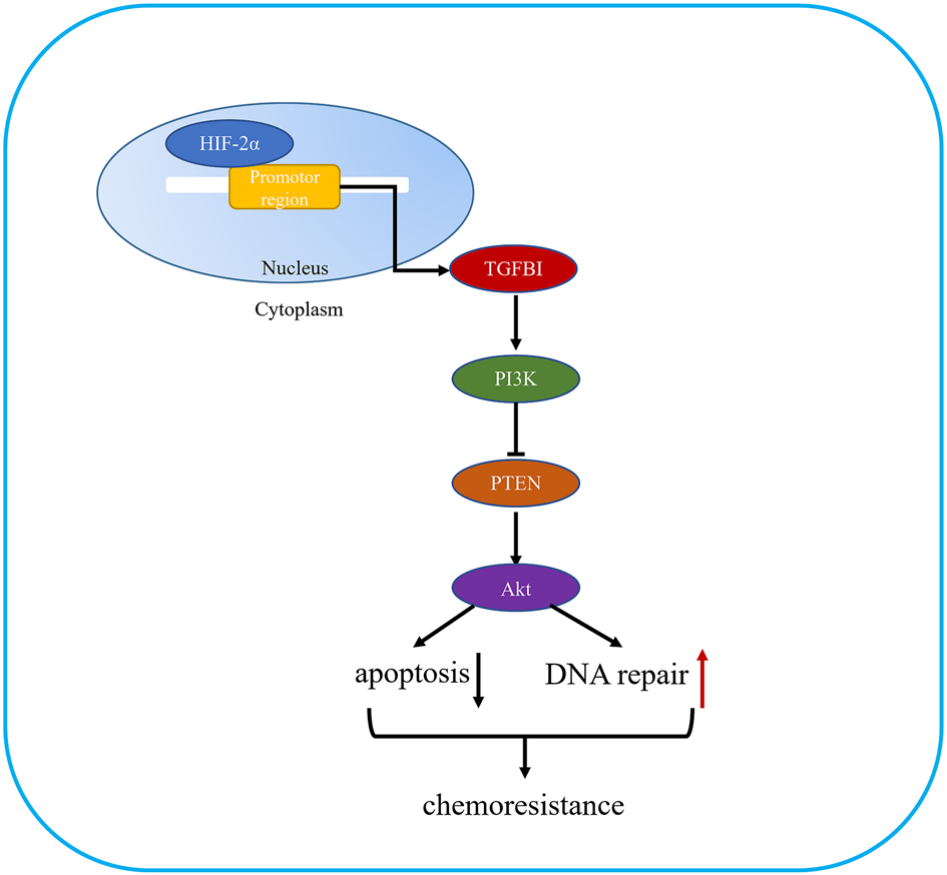
A model of the role of TGFBI in chemoresistance.

Moreover, TGFBI was selected to further explore its specific mechanism in hypoxia and ovarian cancer progression and chemotherapy. Our results showed that TGFBI was up-expressed under hypoxic condition and HIF-2α overexpression, but remained unchanged when HIF-1α was overexpressed. And HIF-2α binding site was found in the promoter region of TGFBI, indicating HIF-2α could directly promote TGFBI transcription. Additionally, we found that the expression level of TGFBI may be positively related with the IC_50_ of platinum including oxaliplatin. Moreover, TGFBI was highly expressed in cisplatin-resistant ovarian cancer cells than sensitive ovarian cancer cells. The TGFBI expression was increased with the increase of cisplatin concentration gradient, and downregulation of TGFBI could impair the chemoresistance of cisplatin-resistant ovarian cancer cells. Furthermore, we found that TGFBI may function as a key regulator in chemoresistance of ovarian cancer mainly through activating HR DNA repair and NHEJ DNA repair; also, TGFBI activate PI3K/Akt pathway to inhibit cell apoptosis and facilitate chemoresistance (Fig. 6). All in all, TGFBI could serve as a potential target for chemoresistance and its inhibitor may be auxiliary complemented with chemotherapy.

In summary, the hub HRGs were identified and used to establish effective prognosis and diagnosis models for OC diagnosis and prediction. One of the HRGs, TGFBI, was found upregulated by HIF-2α and promoted OC chemoresistance via interfering apoptosis and facilitating DNA damage repair through the activation of PI3K/Akt pathway, further verifying the effectiveness of the bioinformatic strategy utilized here.

## Methods and materials

### Data acquisition of HRGs related with OC

The mRNA expression and corresponding clinicopathologic data associated with OC were retrieved from The Cancer Genome Atlas (https://portal.gdc.cancer.gov/)- Ovarian Serous Cystadenocarcinoma- (TCGA‐OV)^35,36^ (including 379 OV tissue samples and 587 clinical information) as training set. The mRNA expression data of OC tissue samples and related clinical information were retrieved from the Gene Expression Omnibus database (GEO) (https://www.ncbi.nlm.nih.gov/geo/)- GSE63885^37^ (including 70 OV tissues samples and corresponding clinical information) as validation set. A list of HRGs was retrieved from Hallmark gene sets of the Molecular Signatures Database^38^.

### Protein–protein interaction (PPI) network and acquisition of hub HRGs

After PPI analysis by STRING database (https://cn.string-db.org/)^39^, we obtained totally 172 hub HRGs with OV-related clinical characteristics from TCGA.

### Establishment and validation of prognostic predictive models based on hub HRGs

The univariate Cox regression was performed to assess the prognostic value of hub HRGs. Subsequently, the multivariate Cox regression was conducted to identify hub HRGs and the prognostic HRGs classifier was constructed. The regression coefficient (β) was derived from multivariate Cox regression analysis^40^. The composition of the final HRGs classifier was selected to generate the risk score based on the following formula:

$$\begin{aligned} Prognosis\;Index & = \left( {\upbeta {\text{gene}}1{\text{*expression}}\;{\text{level}}\;{\text{of}}\;{\text{gene}}1} \right) + \left( {\upbeta {\text{gene}}2{\text{*expression}}\;{\text{level}}\;{\text{of}}\;{\text{gene}}2} \right) + \ldots \\ & \quad + \left( {\upbeta {\text{geneN*expression}}\;{\text{level}}\;{\text{of}}\;{\text{geneN}}} \right) \\ \end{aligned}$$

All patients were divided into two groups (high-risk and low-risk group) based on the median value of the risk score. The risk score of the validation cohort was also calculated using the same formula.

### Survival analysis

Based on Kaplan–Meier analysis, overall survival (OS) was compared between groups with high- and low-risk. To validate the predictive accuracy of the risk models, receiver operating characteristic (ROC) curve was generated by using the R language package “timeROC”^41^.

### Gene set enrichment analysis (GSEA)

Gene set enrichment analysis (GSEA)^42^ was conducted to determine underlying mechanisms of hub HRGs classifier on OC with “Clusterprofiler” package^43^.

### Estimation of immune cell type fractions and immune-related genes

CIBERSORT^44^ was performed to characterize the cell composition and immune cells infiltration. The fractions of immune cell types between high-risk and low-risk groups were assessed by using CIBERSORT.

### Establishment of a diagnostic predictive signature based on hub HRGs

The diagnostic prediction model was constructed to predict OC based on hub HRGs effectively. Sensitivity, specificity, AUC, and 95% CI were calculated to assess the accuracy of the prediction model. The model is as follows:

$$\log it\left( P \right) = {\upbeta }0 + {\upbeta }1x1 + {\upbeta }2x2 + \ldots + {\beta M}x{\text{M}}$$

### Cell culture

Normal human ovarian epithelial cell line IOSE80 and five human ovarian cancer cell lines (3AO, A2780, Caov3, OVCAR3 and SKOV3) were from ATCC, Shanghai Cell Bank of Chinese Academy of Sciences and Shandong Academy of Medical Sciences. The cisplatin-resistant derivate ovarian cancer cell line A2780/CDDP was cultured in intermittent incremental exposure to cisplatin. The cells were cultivated in 1640 supplemented with 10% fetal bovine serum (FBS) at 37 °C with 5% CO_2_. Hypoxia was evaluated by treating OC cells with 1% oxygen for 24 h after being cultured under normoxia conditions to 70% confluence.

### Cell transfections

siRNA against TGFBI and negative control siRNA were purchased from RiboBio Co., Ltd (Guangzhou, China). The overexpression of HIF-1α in OC cells was performed using Flag-tagged HIF-1α plasmid and negative control plasmid. The overexpression of HIF-2α in OC cells was performed with lentiviruses carrying EPAS1 or control, purchased from Genechem Co., Ltd. (Shanghai, China).

### Real-time PCR analysis

Total RNA was isolated using Trizol reagent (Invitrogen, Eugene, OR, USA) and following qRT-PCR (Takara) were performed according to the manufacturer’s instruction. The results were normalized to β-actin gene. The following primers were used: H19 F: 5′-TGCTGCACTTTACAACCACTG-3′ and R: 5′-ATGGTGTCTTTGATGTTGGGC-3′; HIF-1α F: 5′-ATCCATGTGACCATGAGGAAATG-3′ and R: 5′-TCGGCTAGTTAGGGTACACTTC-3′; HIF-2α F: 5′-GCGACCATGAGGAGATTCGT-3′ and 5′-GACCGTGCACTTCATCCTCA-3′; TGFBI F: 5′-CTTCGCCCCTAGCAACGAG-3′ and R: 5′-TGAGGGTCATGCCGTGTTTC -3′; AKAP12 F: 5′-ATCTACAGAGAAACCCGAAGAGA-3′ and R: 5′-TGCAGACTTGCTAGGTTCTTTTT-3′; GPC1 F: 5′-TGAAGCTGGTCTACTGTGCTC -3′ and R: 5′-CCCAGAACTTGTCGGTGATGA-3′; and TGM2 F: 5′-CAAGGCCCGTTTTCCACTAAG -3′ and R: 5′-GAGGCGATACAGGCCGATG-3′.

### Western blot analysis

Human ovarian cancer cells with implementations were lysed in RIPA (Beyotime) on ice. Primary antibodies specific to TGFBI (1:1,000, Proteintech), MRP1 (1:100, Santa Cruz Biotechnology), MDR1 (1:100, Santa Cruz Biotechnology), BRAC2 (1:1,000, Cell Signaling Technology), BRCA1 (1:1,000, Cell Signaling Technology), CtIP (1:1,000, Cell Signaling Technology), p-p95 (1:1,000, Cell Signaling Technology), p95 (1:1,000, Cell Signaling Technology), RAD54 (1:1,000, Cell Signaling Technology), RAD51 (1:1,000, Cell Signaling Technology), p-DNA-PKcs (1:1,000, Cell Signaling Technology), DNA-PKcs (1:1,000, Cell Signaling Technology), DNA Ligase IV (1:1,000, Cell Signaling Technology), Artmis (1:1,000, Cell Signaling Technology), Ku80 (1:1,000, Cell Signaling Technology), Ku70 (1:1,000, Cell Signaling Technology), XLF (1:1,000, Cell Signaling Technology), PTEN (1:1,000, Cell Signaling Technology), PI3K-p110 (1:1,000, Cell Signaling Technology), PI3K-p85 (1:1,000, Cell Signaling Technology), p-Akt (1:1,000, Cell Signaling Technology), Akt (1:1,000, Cell Signaling Technology), BCL2 (1:1,000, Cell Signaling Technology), Bax (1:1,000, Cell Signaling Technology), Beclin1 (1:1,000, Cell Signaling Technology) and β-actin (1:2,000, Cell Signaling Technology) were used at 4 °C overnight. After incubation with secondary antibodies (1:1,000, Cell Signaling Technology), immune complexes were detected on Image Lab Software in Molecular Imager ChemiDoc XRS (Bio-Rad).

### Cell adhesion, migration and invasion assays

Cell adhesion assay was measured according to the manufacturers protocol. Transwell assays were performed using a 24-well plate (BD, Corning, NY, USA). Cells (3-5 × 10^5^/well) were seeded in RPMI-1640 without FBS in the top chamber, and were allowed to migrate for 24 to 48 h. The membrane was then washed by PBS for three times and fixed in methyl alcohol for 30 min and stained with crystal violet. Cells were then imaged and counted in five independent fields. Additionally, Matrigel (BD Biosciences) was performed for invasion assay.

### CCK8 assays

Cells (1×10^3^ cells/well) were seeded into a 96-well plate and transfected with specific siRNAs against TGFBI or siNC. Cell Counting Kit-8 was applied to measure the optical density (OD) value at 450 nm based on the manufacturer’s protocol.

### Evaluation of drug sensitivity

Drug sensitivity data of 60 different human cancer cell lines was downloaded from the CellMiner database^45^(https://discover.nci.nih.gov/cellminer/), which including drug sensitivity data (IC_50_ Values). CellMiner contains IC_50_ data for 860 drugs totally. Pearson test was performed to compare the correlation between the TGFBI expression levels and IC_50_ of totally 860 drugs.

### IC_50_ assay

After transfected with specific siTGFBI or siNC, A2780/CDDP cells were seeded on a 96-well plate and cultivated for 24 h for the IC_50_ assay of cisplatin treatment (0, 2.5, 5, 10, 20, 40, 80, 160, 320, 640 µM/L). Cell Counting Kit-8 was applied to measure the OD according to the manufacturer’s protocol.

### Statistical analysis

Continuous variables were performed as the mean ± standard deviation (SD). Differences between groups were measured by two-tailed t-test. P-value < 0.05 was considered statistically significant.

### Supplementary Information

Supplementary Information.

## Data Availability

The data sets analyzed during the current study are available in the TCGA (https://portal.gdc.cancer.gov/), accession numbers TCGA-OV, OV-FPKM. The data used to support the findings of this study are available from the corresponding author upon request.
