# Supplementary material for: HIF-2α-dependent TGFBI promotes ovarian cancer chemoresistance by activating PI3K/Akt pathway to inhibit apoptosis and facilitate DNA repair process
Source: Sci Rep. 2024 Feb 16;14:3870. doi: 10.1038/s41598-024-53854-y (PMC10873328; doi:10.1038/s41598-024-53854-y)
Supplement: Supplementary file 1 — Supplementary Information. [file 41598_2024_53854_MOESM1_ESM.docx]

Supplementary Material

HIF-2α-dependent TGFBI promotes ovarian cancer chemoresistance by activating PI3K/Akt pathway to inhibit apoptosis and facilitate DNA repair process

Sijia Ma^1,2,3^, Jia Wang^1^, Zhiwei Cui^1^, Xiling Yang^1,2,3^, Xi Cui^1,2,3^, Xu Li^1,2,3^, Le Zhao^1,2,3,*^

^1^Department of Obstetrics and Gynecology, the First Affiliated Hospital of Xi’an Jiaotong University, Xi’an, Shaanxi 710061, P.R. China

^2^Center for Translational Medicine, the First Affiliated Hospital of Xi’an Jiaotong University, Xi’an, Shaanxi 710061, P.R. China

^3^Key Laboratory for Tumor Precision Medicine of Shaanxi Province, the First Affiliated Hospital of Xi’an Jiaotong University, Xi’an, Shaanxi 710061, P.R. China

*** Correspondence:**Corresponding Author: Le Zhao,
email: [zhaole2@mail.xjtu.edu.cn](mailto:zhaole2@mail.xjtu.edu.cn)

**Supplementary Figures**
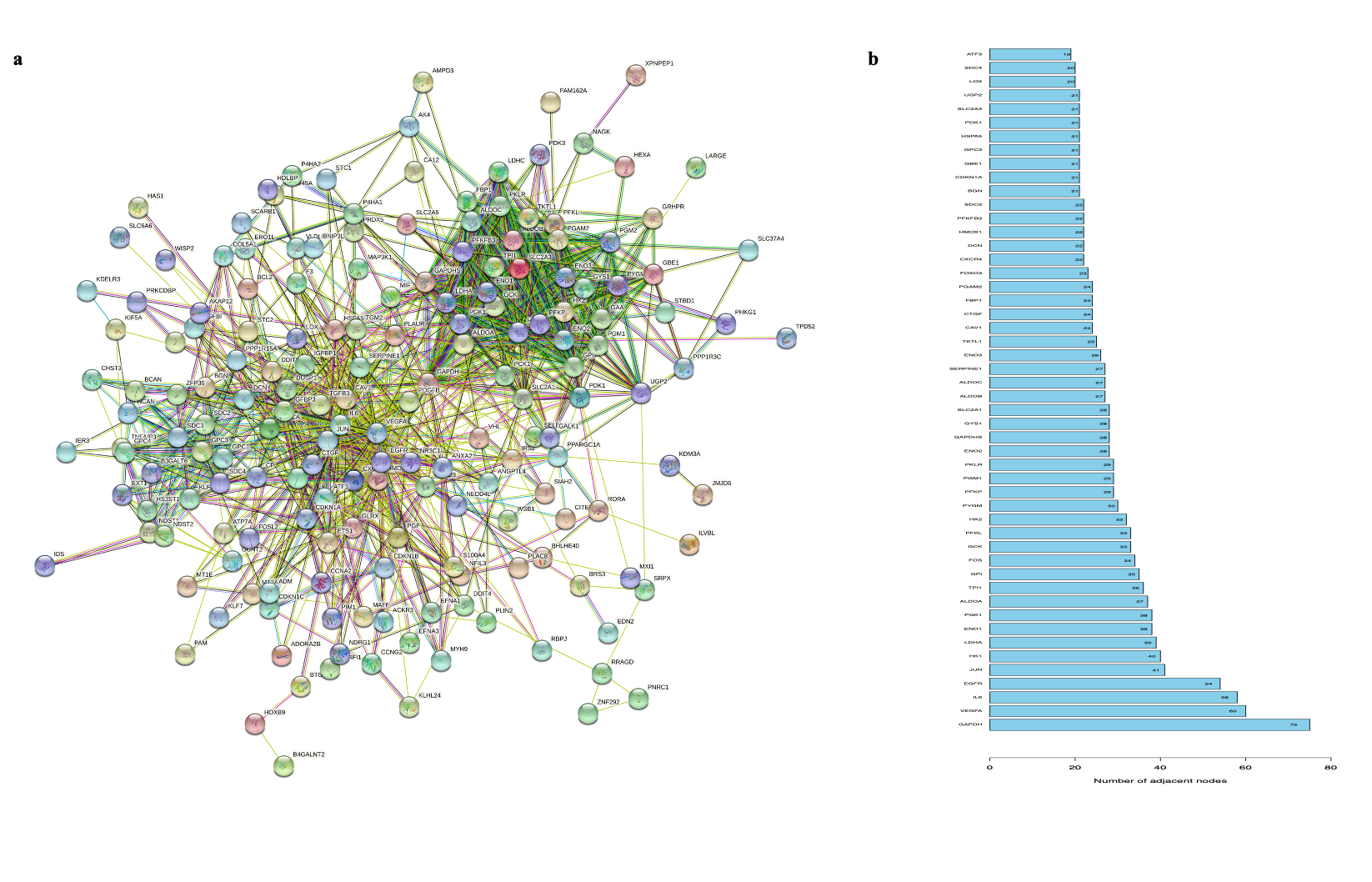


Supplementary Figure 1. The protein-protein interaction analysis of hypoxia-related genes. a. The PPI analysis of 179 hypoxia-related genes. b. The top 50 genes with highest number of adjacent nodes.


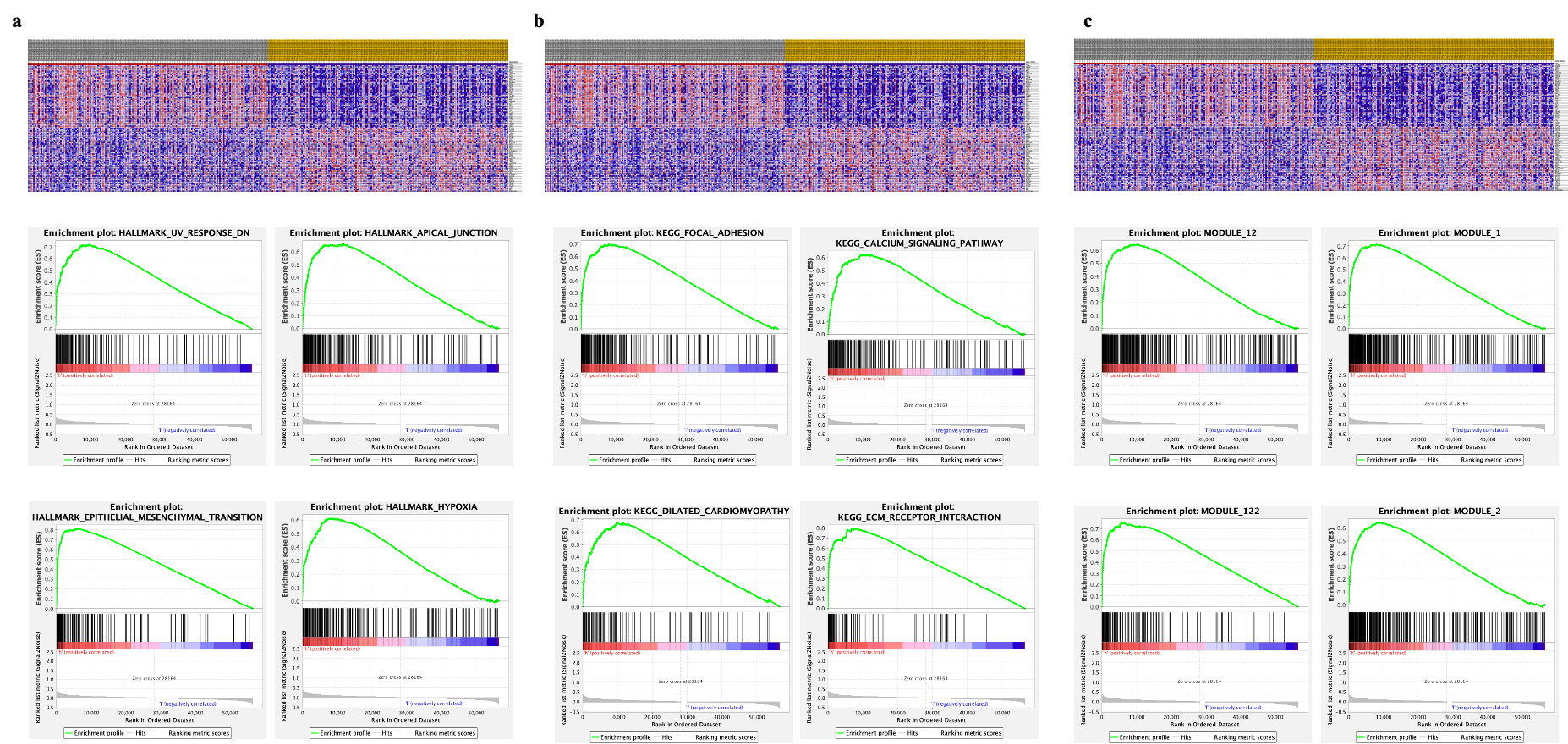


Supplementary Figure 2. Enrichment plots of activated signaling pathways in the high hypoxia risk group, including Hallmarks analysis (2a), KEGG analysis (2b) and GO analysis (2c).


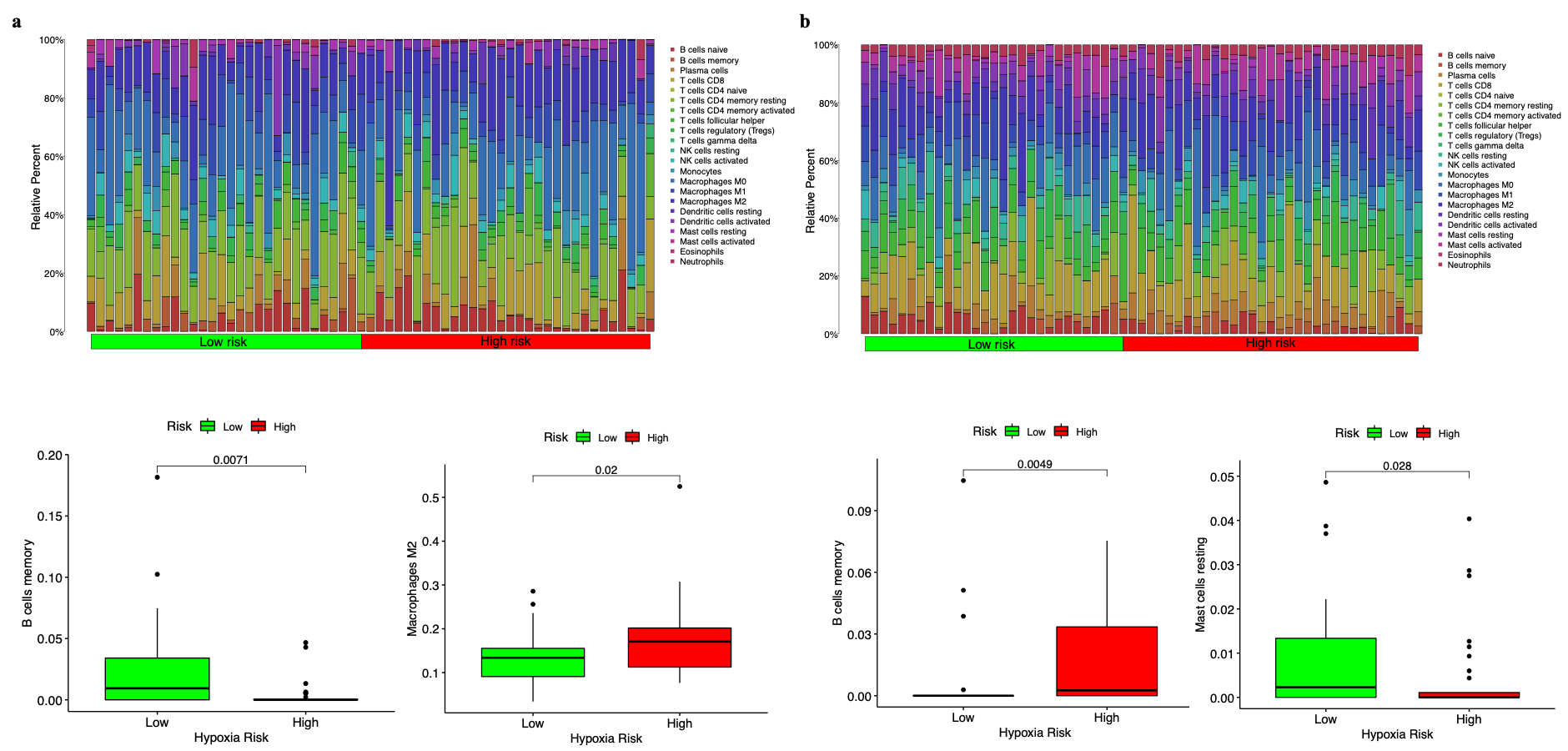


Supplementary Figure 3. Immunity analysis between the high and low hypoxia risk groups. 3a. The heatmap of all immune cells and significantly differential immune cells in training cohort. 3b. The heatmap of all immune cells and significantly differential immune cells in validation cohort.


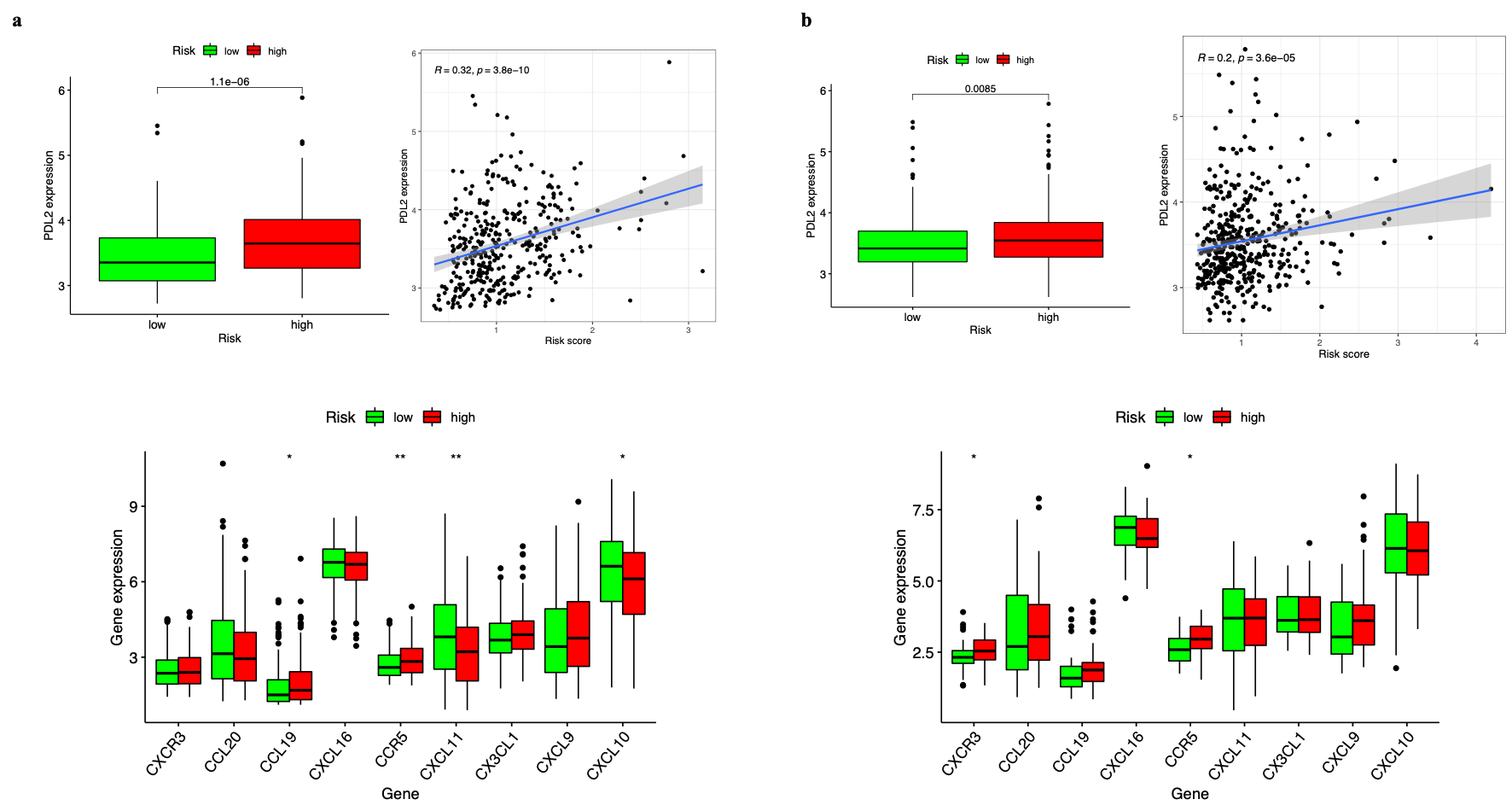


Supplementary Figure 4. Expression of immune-related genes in the high and low hypoxia risk groups. 4a. Boxplots of genes that negatively regulated cancer immune cycle in the high and low hypoxia risk groups in training cohort. 4b. Boxplots of genes that negatively regulated cancer immune cycle in the high and low hypoxia risk groups in validation cohort.


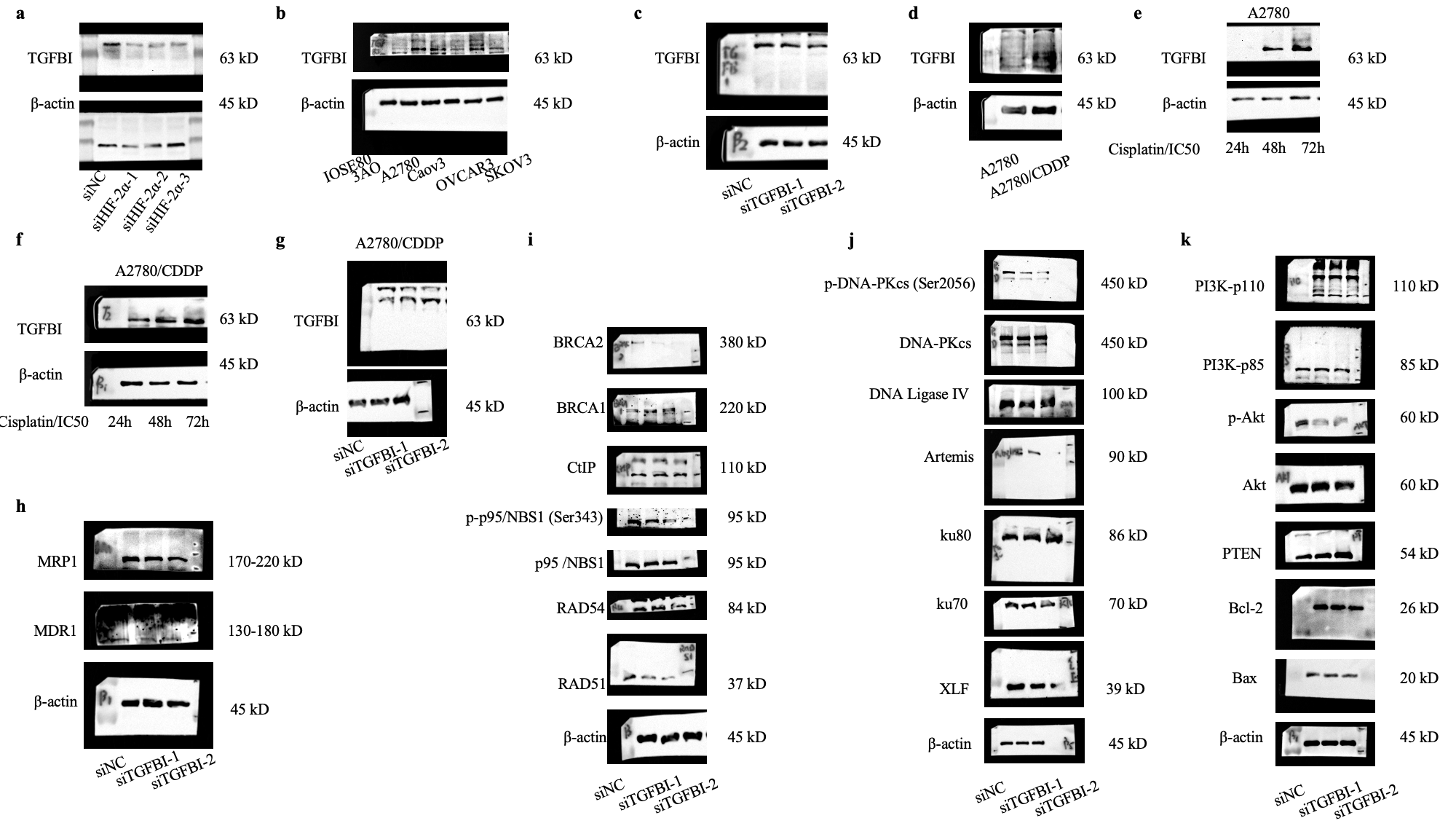


Supplementary Figure 5. The uncropped images of all Western blots. 5a. The protein expression level of TGFBI after transfected with siHIF2a. 5b. The protein expression level of TGFBI in normal ovarian cell line and 5 ovarian cancer cell lines. 5c. The protein expression level of TGFBI after knocking down by si-RNA interfere. 5d. The protein expression level of TGFBI in cisplatin-sensitive ovarian cancer cell line-A2780 and cisplatin-resistant ovarian cancer cell line-A2780/CDDP. 5e. The protein expression level of TGFBI after time gradient of cisplatin in A2780 (IC50 as 119.2μM). 5f. The protein expression level of TGFBI after time gradient of cisplatin in A2780/CDDP (IC50 as 258.8μM). 5g. The protein expression level of TGFBI after knocking down by si-RNA interfere in A2780/CDDP. 5h. The protein expression level of MRP1 and MDR1 in A2780/CDDP after TGFBI knockdown. 5i. The protein expression level of Homologous Recommendation DNA repair related markers in A2780/CDDP after TGFBI knockdown. 5j. The protein expression level of Non-Homologous End Joining (NHEJ) DNA repair related markers in A2780/CDDP after TGFBI knockdown. 5k. The protein expression level of PTEN, PI3K/p110, PI3K/p85, p-Akt, Akt, BCL2, Bax and Beclin1 in A2780/CDDP after TGFBI knockdown.

**Supplementary Tables**

Supplementary Table 1. The prognosis Index of prognosis model.

| id | coef | HR | HR.95L | HR.95H |
| --- | --- | --- | --- | --- |
| TGFBI | 0.00511378 | 1.00512688 | 0.99903924 | 1.01125161 |
| CDKN1B | 0.01957213 | 1.01976492 | 1.00886456 | 1.03078306 |
| AKAP12 | 0.02298549 | 1.02325169 | 1.00534459 | 1.04147776 |
| GPC1 | 0.01006978 | 1.01012065 | 0.99893031 | 1.02143635 |
| TGM2 | 0.00514497 | 1.00515823 | 1.00051306 | 1.00982497 |
| ANGPTL4 | 0.01678315 | 1.01692478 | 1.00333771 | 1.03069585 |

Supplementary Table 2. The area under the curve of four diagnostic models.

| Test Result Variable(s) | Area | Std. Errora | Asymptotic Sig.b | Asymptotic 95% Confidence Interval |  |
| --- | --- | --- | --- | --- | --- |
|  |  |  |  | Lower Bound | Upper Bound |
| Predicted Probability-1 | .645 | .108 | .117 | .434 | .856 |
| Predicted Probability-2 | .826 | .061 | .000 | .706 | .946 |
| Predicted Probability-3 | .875 | .057 | .000 | .762 | .987 |
| Predicted Probability-4 | .893 | .047 | .000 | .801 | .986 |

a Under the nonparametric assumption

b Null hypothesis: true area = 0.5

Supplementary Table 3. The Variables in the Equation of four diagnostic models.

|  |  | **B** | **S.E.** | **Wald** | **df** | **Sig.** | **Exp(B)** |
| --- | --- | --- | --- | --- | --- | --- | --- |
| **Step 1a** | TGFBI | -.002 | .001 | 7.775 | 1 | .005 | .998 |
|  | Constant | 4.680 | .483 | 93.714 | 1 | .000 | 107.791 |
| **Step 2b** | TGFBI | -.003 | .001 | 11.010 | 1 | .001 | .997 |
|  | GPC1 | .021 | .007 | 8.743 | 1 | .003 | 1.021 |
|  | Constant | 4.138 | .486 | 72.445 | 1 | .000 | 62.708 |
| **Step 3c** | TGFBI | -.003 | .001 | 11.820 | 1 | .001 | .997 |
|  | AKAP12 | -.006 | .002 | 6.807 | 1 | .009 | .994 |
|  | GPC1 | .022 | .008 | 8.460 | 1 | .004 | 1.023 |
|  | Constant | 4.897 | .657 | 55.476 | 1 | .000 | 133.914 |
| **Step 4d** | TGFBI | -.005 | .001 | 11.669 | 1 | .001 | .995 |
|  | AKAP12 | -.007 | .002 | 9.311 | 1 | .002 | .993 |
|  | GPC1 | .022 | .008 | 7.043 | 1 | .008 | 1.023 |
|  | TGM2 | .022 | .009 | 5.774 | 1 | .016 | 1.022 |
|  | Constant | 4.699 | .730 | 41.402 | 1 | .000 | 109.853 |

a Variable(s) entered on step 1: TGFBI.

b Variable(s) entered on step 2: GPC1.

c Variable(s) entered on step 3: AKAP12.

d Variable(s) entered on step 4: TGM2.
